# Why don't health workers prescribe ACT? A qualitative study of factors affecting the prescription of artemether-lumefantrine

**DOI:** 10.1186/1475-2875-7-29

**Published:** 2008-02-05

**Authors:** Beatrice Wasunna, Dejan Zurovac, Catherine A Goodman, Robert W Snow

**Affiliations:** 1 Eastern and Southern Africa Centre of International Parasite Control (ESACIPAC)/KEMRI, P.O. Box 54840-00200, Nairobi, Kenya; 2 Malaria Public Health & Epidemiology Group, Centre for Geographic Medicine Research-Coast, Kenya Medical Research Institute/Wellcome Trust Research Programme, P.O. Box 43640, 00100 GPO, Nairobi, Kenya; 3 Centre for Tropical Medicine, University of Oxford, John Radcliffe Hospital, Headington, Oxford, OX3 9DU, UK; 4 Department of International Health, School of Public Health and Center for International Health and Development, 5^th ^Floor, Boston University School of Public Health, 85 East Concord Street, Boston, MA 02118, USA; 5 London School of Hygiene and Tropical Medicine, Health Policy Unit, Keppel Street, London, WC1E 7HT, UK

## Abstract

**Background:**

Kenya recently changed its antimalarial drug policy to a specific artemisinin-based combination therapy (ACT), artemether-lumefantrine (AL). New national guidelines on the diagnosis, treatment and prevention were developed and disseminated to health workers together with in-service training.

**Methods:**

Between January and March 2007, 36 in-depth interviews were conducted in five rural districts with health workers who attended in-service training and were non-adherent to the new guidelines. A further 20 interviews were undertaken with training facilitators and members of District Health Management Teams (DHMTs) to explore reasons underlying health workers' non-adherence.

**Results:**

Health workers generally perceived AL as being tolerable and efficacious as compared to amodiaquine and sulphadoxine-pyremethamine. However, a number of key reasons for non-adherence were identified. Insufficient supply of AL was a major issue and hence fears of stock outs and concern about AL costs was an impediment to AL prescription. Training messages that contradicted the recommended guidelines also led to health worker non-adherence, compounded by a lack of follow-up supervision. In addition, the availability of non-recommended antimalarials such as amodiaquine caused prescription confusion. Some health workers and DHMT members maintained that shortage of staff had resulted in increased patient caseload affecting the delivery of the desirable quality of care and adherence to guidelines.

**Conclusion:**

The introduction of free efficacious ACTs in the public health sector in Kenya and other countries has major potential public health benefits for Africa. These may not be realized if provider prescription practices do not conform to the recommended treatment guidelines. It is essential that high quality training, drug supply and supervision work synergistically to ensure appropriate case management.

## Background

The World Health Organization (WHO) recommends that efficacious artemisinin-based combination therapies (ACTs) should be the preferred replacements for failing monotherapies, such as sulphadoxine-pyrimethamine (SP) [1,2]. The Global Fund for AIDS, TB and Malaria is currently spending millions to fund ACT policy implementation across Africa. It is, therefore, imperative that these highly effective drugs reach their target audience. Kenya implemented a change in its first-line treatment policy for uncomplicated malaria from SP to the ACT artemether-lumefantrine (AL) in 2006 [3]. AL was supplied by the Kenyan Ministry of Health (MoH) to all government health facilities within the district health system – starting from the lowest level dispensaries, to mid-level health centres and the higher level district hospitals. Outpatient care in dispensaries and health centres is most commonly provided by nurses, while clinical officers are usually outpatient providers in hospitals. At all government facilities, AL should be provided to patients free of charge. AL is an expensive medicine in the private sector, costing from 600 to 680 Kenya Shillings (equivalent to 9–10 US$), putting it beyond the reach of most households. Government provision is, therefore, the main delivery channel to the majority of the poor.

Alongside AL delivery to health facilities, the key implementation activities during 2006 included in-service training for health workers, and development and dissemination of national malaria guidelines. The training was organized in a cascade manner, starting at the national level by training provincial trainers who then trained district trainers who further trained approximately 9,000 health workers [3]. The training was organized outside health facilities, in the form of 3-day workshops for approximately 30 participants per training course. These 3-day workshops were facilitated by the District Health Management Teams (DHMTs). One day was devoted to the management of uncomplicated malaria, and teaching modalities included lectures and theoretical case scenarios, but no clinical practice. In support of the implementation of the new drug policy, a concerted effort was made to harmonize national case-management guidelines and training materials [4-6], which were provided to health workers during the-in-service training sessions.

Between October and December 2006, four to six months after the policy was deemed rolled out, a facility-based assessment was undertaken to evaluate the extent to which febrile patients were managed in accordance with the revised guidelines in government health facilities [7]. The most important observation of this descriptive study was that despite AL being in stock on the day of the survey only 28% of 866 children needing treatment with AL according to national guidelines were prescribed this drug. Even of those children seen by health workers who had attended in-service training, only 43% meeting the same criteria were prescribed AL. An even more worrying pattern has been observed in older children and adults where only 34% of patients seen by trained health workers who diagnosed malaria at facilities with AL in stock were prescribed AL. This clearly raised concern about effective implementation of the new drug policy and prompted a more detailed qualitative investigation on why health workers might elect not to prescribe AL. These findings are reported in this paper.

## Methods

### Malaria treatment guidelines

The new recommendations state that all febrile children below five years of age and above 5 kg in high malaria risk areas should be presumptively treated with AL. In low malaria risk areas, absence of another obvious cause of fever is used as criterion for treatment. All parts of Kenya are classified as high malaria risk areas, except for the highlands of Central and Nairobi provinces. In patients five years of age and older seen at health facilities where malaria diagnostics (microscopy or rapid diagnostic tests) are available, all febrile patients without another obvious cause of fever should have a malaria test performed, and health workers should treat with AL when the malaria test is positive. At health facilities where malaria diagnostics are not available, all febrile patients should be presumptively treated with AL in the absence of another obvious cause of fever.

Compared with conventional monotherapies, AL has a more complex six-dose treatment schedule over three days. The total number of tablets and packaging of AL differs between four weight categories. Furthermore, lumefantrine has poor oral bioavailability; absorption varies considerably among individual doses and between patients, and is significantly reduced in the acute phase of malaria [8-10]. Thus, introduction of AL into clinical practice requires a greater effort to ensure adherence to diagnostic, prescription, drug dispensing and counseling recommendations. The guidelines, therefore, specify counseling and drug administration tasks that health workers should perform when prescribing AL, including counseling patients on the dosing schedule, importance of a fatty diet, what to do in case of vomiting, and administration of the first dose under the health worker's supervision.

### Study area

The study was conducted in five rural districts of variable intensities of malaria transmission (Kwale, Kisii, Gucha, Bondo and Makueni) between January and March 2007. The study districts, and the structure and provision of clinical services for treating uncomplicated malaria have been described previously [11,12]. For clinical management purposes all districts are classified as high malaria risk areas and the same malaria case management recommendations apply in all five districts [5]. This qualitative study was an extension of a larger quantitative study evaluating the use of AL under operational conditions, which was conducted in all government health facilities within the five districts [7].

### Sampling of health workers

The quantitative study, undertaken between October and December 2006, identified 227 health workers located in 193 government health facilities. These health workers formed the basis of the sampling frame for the present study. Four criteria were applied to these health workers to qualify for inclusion in this qualitative study: 1) they must have received training on the new treatment guidelines; 2) they were working at health facilities where AL was in stock on the day of the survey; 3) they were routinely involved in the diagnosis of malaria at their facility; and 4) during the 2006 facility-based assessment they prescribed AL for less than 40% of patients for whom they made a routine diagnosis of malaria. This group was selected deliberately because of their degree of non-adherence, and the lack of other obvious reasons for this. 84 health workers met the first three criteria and 36 met all four criteria. Of these 36, four did not prescribe AL to any patients during the assessment, with the remainder prescribing to some but not others. The 36 health workers were all identified and agreed to participate in the qualitative study. Most of the health workers were nurses (89%). Only four out of the 36 health workers (11%) were clinical officers. Clinical officers are medical assistants with three years of training and perform medical duties similar to those of doctors, with the exception of surgical procedures. Their ages ranged from 25 to over 55 years. Of the 36 health workers most (78%) were working in dispensaries. The other eight were working in health centres (six) and district hospitals (two) (Table 1).

**Table 1:** Characteristics of health workers stratified by cadre, age, gender and health facility type.

	**Number**	**Percentage**
**Number of respondents**	36	
**Number of districts**	5	
**Cadre**		
Clinical Officers	4	11%
Nurses	32	89%
**Age**		
<25	-	
25–34	10	28%
35–44	12	33%
45–55	13	36%
>55	1	3%
**Gender**		
Male	21	58%
Female	15	42%
**Health facility type**		
District hospital	2	5%
Health Centre	6	17%
Dispensary	28	78%

### Data collection methods

Since the study was addressing the sensitive topic of non-adherence to guidelines, individual in-depth interviews (IDIs) were selected as the most appropriate data collection tool to investigate opinions and reasons underlying such behaviour. A semi-structured interview guide was developed which allowed for flexibility within the discussions, and explored health worker perceptions about the new treatment policy and reasons underlying their decisions not to prescribe AL. The influence of perceived severity of illness on prescribing practices was explored through a case vignette methodology in which health workers were asked to respond to a specific case management scenario. The guide was piloted in a rural non-study district (Kirinyaga). Since the qualitative study was an extension of the quantitative survey the study was introduced to health workers as a follow up of findings of the preceding quantitative malaria case management study. Focusing questions on reasons for non-adherence only could have led health workers to feel defensive and be less open in their responses. This was avoided by combining these questions with those on general health worker characteristics, in-service training on the new treatment policy and health worker perceptions of AL, in order to put respondents at ease.

The health worker (HW) interviews were augmented with additional interviews with key informants responsible for implementing the new treatment policy in the district including training facilitators (TF) (10) and DHMT members (10). Areas specifically covered with key informants included the content of the malaria case-management messages communicated to training participants, drug supply and programmatic constraints in the implementation of AL in the districts. All IDIs were conducted by one of the authors (BW) in English and tape recorded for subsequent coding and analysis. Each session lasted from 45 minutes to one hour 30 minutes.

### Data analysis

Information recorded on tape during each IDI was transcribed and subjected to content analysis using N-Vivo, version 7 qualitative text analysis software (QSR International, Southport, United Kingdom) [13,14]. The process of analysis involved familiarization with the data, development of initial codes based on the research questions and issues emerging from the data, refinement of codes, and their allocation to broad themes. Data obtained from health workers, training facilitators and DHMT members were compared for purposes of triangulation. For example, responses on training messages were triangulated between health workers and training facilitators. This process of triangulation ensured completeness and validity of the findings from each source.

### Ethical considerations

Ethical approval was obtained from the KEMRI/National ethical review committee (KEMRI SSC number 1197). Before each interview the study objectives, method to be used (tape recording) and the voluntary nature of participation was explained to each interviewee and informed written consent was obtained before the interview began.

## Results

The potential factors leading to provider non-adherence emerging from the in-depth interviews have been organized into eight broad themes: health worker perceptions of AL; concerns over cost; fear of stock-outs; excess stocks of non-recommended antimalarials; ambiguous training messages; perceived severity of illness; patient pressure to obtain certain types of anti-malarial; and health system weaknesses (staffing versus work load and supervision).

### Health worker perceptions of AL

It was expected that health workers' negative perceptions of AL would be a key reason for non-prescription. However, the data showed that AL was perceived as an efficacious treatment compared to SP by nearly all health workers, and most perceived that the numbers of revisits had declined since the introduction of AL. Safety was also not a concern. The majority of health workers reported that they had not received any cases of patients with side effects from AL. They further implied that they perceived AL as having a higher tolerability compared to amodiaquine and quinine.

With regard to the dosing schedule for AL, most health workers felt this was not complex, asserting that the three-day period was similar to that of amodiaquine and, therefore, patients were comfortable with the duration. Health workers further stated that there were many other drugs such as antibiotics that were taken over a similar or longer duration. However, other health workers were of the opinion that the number of tablets taken per day and the dosing schedule was cumbersome and likely to compromise compliance. They suggested that a shorter duration and fewer tablets were likely to improve patient compliance to the treatment regimen.

### Concern over AL cost

Health workers were of the opinion that AL was expensive, despite the appreciation that the government had provided it free at their health facilities. Some stated that, because it was an expensive drug, they usually restricted prescription. Health workers had concerns regarding whether the government would be able to sustain the supply of AL. One health worker from Makueni District suggested that the introduction of generics might be a possible solution to reduce costs:

"*Can the government ensure the consistent supply of the drug? The way the drug is being supplied at the moment, I have doubts that the government will sustain the supply of Coartem (brand name for AL) unless we bring other generics". (HW, Makueni)*

### Fear of stock-outs

Fear of stock-outs was reported in all five study districts. Health workers reported that the supply of AL had been inconsistent during the initial stages of implementation of the new policy and there were shortages of some dosages, particularly those for adults. Three of the five districts (Bondo, Makueni and Gucha) had experienced at least one month of AL stock-out during the three-month period between December 2006 and February 2007. Nearly all health workers indicated that they were rationing the drug because they were not certain of the next supply based on previous stock-outs periods.

"*You may run out of it (AL) and the supply is not certain. In fact the number of doses we receive is not commensurate to the number of clients that we see. We are always sure that we will run short of it in due course. That's why we try to ration". (HW, Bondo)*

As a result, health workers aimed to target the drugs to those patients they perceived as most in need or most "deserving".

"*It is very true because if we were to give AL and the dosages are limited it could reach a point where you are required to give AL and you don't have it and you used it on the wrong patient. Part of the reasoning is that on a daily basis we are giving the drug to those who 'deserve'. Therefore some will not get AL and others will. I don't have to give someone who doesn't 'deserve' it and I always insist on that". (HW, Makueni)*

There were also concerns that there might be seasonal stock-outs at times when there might be an increase in malaria cases.

### Excess stocks of non-recommended antimalarials

DHMT members and health workers in Bondo, Makueni, Kisii and Gucha reported having received a large supply of amodiaquine from the Kenya Medical Supplies Agency (KEMSA) between the months of December 2006 and February 2007, in contrast to the inadequate supplies of the recommended AL. This created a difficult position for some health workers who struggled to understand when to prescribe AL and how to manage the continued supply of amodiaquine.

*"Actually it was late last month *(January, 2007)*and early this month *(February, 2007)*, that they supplied us with both AL and amodiaquine. So there is that element of confusion to the health workers. Health workers have complained about being supplied with amodiaquine in large quantities despite the fact that they are also getting AL. They are asking why the government is still supplying so much of it, if they are not supposed to use?". (DHMT, Makueni)*

These concerns were largely directed to KEMSA.

"I thought this supply was to be wiped out from KEMSA and all central supply should be having it cut off if it will not be required. If they are supplied, it means you use it otherwise why is it being supplied? I'm sure the people from KEMSA know about AL". (DHMT, Bondo)

The DHMT members added that unless the government addressed the problem of amodiaquine supply, health worker prescriptions of AL were likely to be compromised.

### Training messages

Some of the key messages delivered during training influenced health workers' prescribing decisions. Incorrect messages were reportedly received, for example that compulsory parasitological testing was required before prescribing AL, and that amodiaquine was still effective. Around half of the HWs stated these messages as reasons for not prescribing AL.

It was widely reported by both those receiving and providing training that there was a key emphasis during in-service training on obtaining confirmed parasitic diagnosis using microscopy or rapid diagnostic tests (RDTs) before prescribing AL.

*"In the first place when we got this AL we were told not to use them unless we get those kits *(RDTs)*". (HW, Kwale)*

"*We said they have to test before you put the patient on the drug (AL) and the test has to be positive. We are also encouraging the use of RDTs even at the dispensary level. So we said there is no reason for not testing because in the event that you do not have a microscope, you can use the RDT. That one we emphasized, that you have to be tested before prescribing AL". (Training facilitator (TF), Bondo)*

DHMT members indicated that this restriction had prevented many health workers from prescribing AL because they were waiting for RDTs to be supplied to their health facilities, particularly to those without microscopy.

The importance of confirmed diagnosis was particularly emphasized for patients of five years and above. For this age group health workers reported being told it was compulsory to test before prescribing AL, regardless of the availability of diagnostics. Only a few health workers said that they could treat presumptively with AL if diagnostics were not available. They, therefore, often defaulted to using monotherapies for older patients.

"We were told that "we don't give Coartem (AL) before testing" for patients over five years and adults, so our drug of choice remains as SP, amodiaquine and quinine. Coartem doses for patients over five and adults are all in the stores as we wait for the RDTs". (HW, Kwale)

In Makueni, health workers reported that they had been told by training facilitators that amodiaquine was still effective and they could, therefore, still prescribe it (this was confirmed in interviews with training facilitators).

"*We were told that amodiaquine still can cure malaria because 92% of the patients get cured when using amodiaquine, while with Coartem it is 96%. That is why we are still using them". (HW, Makueni)*

Most health workers reported that they were told by training facilitators to treat all childhood fevers presumptively as malaria using AL, in accordance with algorithms developed in the national guidelines and harmonized with the Integrated Management of Childhood Illness (IMCI) fever algorithms. However, some health workers reported that they had been told by training facilitators to rule out other diagnoses before prescribing AL in febrile children. They were, therefore, employing some degree of clinical judgement.

"What we were telling them, is that when a child comes with fever, you rule out it is not fever as a result of other illness, that is they were to treat as per IMCI guidelines. However, we told them every person must be tested to rule out that is not malaria, and then if it is meningitis or if it is bacterial infection they can treat according to the IMCI guidelines. They should not treat fever as malaria". (TF, Kisii)

### Perceived severity of illness

The vignette case scenario sought to explore whether the health worker was more inclined to prescribe AL to a child <5 years with high temperature (40°C) or to a child with history of fever but a currently normal temperature and why. Following from the vignette most health workers reported that they would prescribe AL to the patient who had a high temperature but not to those with only history of fever.

"*When a patient comes here (health facility), I check the temperature and if the patient has fever. I do not have a lab here so I have to give the patient who deserves. The fever has to be high. So in this story I will give the girl with 37°C amodiaquine because her fever is not too high maybe there is another illness so I will investigate further and the other child with temperature of 40°C I will give AL because the fever is high". (HW, Kisii)*

### Patient pressure to obtain a certain type of anti-malarial

Patient pressures were reported by some health workers as a reason for not prescribing AL. A few health workers reported that some patients preferred SP to AL because SP was taken as a single dose, and they could then forget about the treatment.

Many patients were reported to prefer injections which were available for quinine, but not AL. In Kwale and Kisii districts health workers reported that some patients preferred injections and if they were not given one they felt they had not received any treatment.

Only two health workers stated that mothers preferred amodiaquine syrup to AL because they found the six-tablet pack cumbersome. The health workers suggested that an AL suspension/syrup should be provided for children.

*"If we can get suspensions for AL because the under fives are the most that suffer from malaria. You know the mothers are used to amodiaquine syrup so they usually complain that they have to crush too many tablets". (HW, Makueni)*.

### Work load and supervision constraints

Under-staffing in the rural health facilities was also reported as a barrier to adherence to the new treatment policy. Both health workers and DHMT members indicated that the shortage of staff had resulted in increased workload thereby affecting the delivery of quality care particularly in the rural health facilities. DHMT members further reported that staff retention was a challenge since some of the health workers who had been trained on the new guidelines had moved to other facilities leaving untrained health workers at the facilities. Staffing issues were seen as a particular concern in AL prescription because of the additional time required for counseling, and direct observation of the first dose, record keeping, and confirmed diagnosis for patients over five in facilities with diagnostics.

"We were told to give quality care, it is one thing saying but another doing. Sometimes you just give quantity care because I'm all alone here. I have 2–3 community health workers and they are not conversant with the new treatment policy. Imagine I am having seven antenatal mothers, six clients of family planning, fifteen under fives and ten adults. I am the only health worker and in the prescription of AL it is recommended that one should take time with that patient so that you can come up with a good diagnosis, so sometimes I feel harassed. If there could be additional health workers, then we can give the patient quality care". (HW, Kwale)

Health workers reported that there had been no supervisory visits following training on the new guidelines. The DHMT indicated that the inadequacy of supervisory visits was entirely due to a lack of transport and human resources to cover the entire district. They further recognized that improved supervision could have corrected any misinformation conveyed during in-service training.

## Discussion

Prescribers' adherence to guidelines is critical for the successful implementation of any new drug policy. However, poor adherence is well described in areas of child health, family planning and diabetes [15-19]. Discordance between the observed behaviour of health professionals and national standard treatment guidelines for uncomplicated malaria has been documented in Kenya [12,20], Malawi [21], Uganda [22,23], Benin [24], Central African Republic [25], Ghana [26] and Nigeria [27]. A series of studies in low and middle-income countries have assessed the relationship between health worker factors, patient-client factors, health facility environment, administrative environment and socio-cultural environments on health worker performance [28-32]. However, little in-depth qualitative investigation of the reasons behind these associations (or lack of associations) has been undertaken. This study has addressed this gap for the case of prescription of ACTs, by investigating health workers' perceptions and understanding of the new antimalarial treatment policy, and reasons underlying their non-adherence to the national antimalarial treatment guidelines.

Health workers generally have a positive perception of AL, which they see as more efficacious and more tolerable than previously used antimalarials such as SP and amodiaquine. Adherence to AL has been suggested as a major constraint to its effectiveness [33]. However, only a few health workers were concerned about the complexity of the dosing schedule. Most did not see this as a cause for non-prescription, as antibiotics and other antimalarials, with the exception of SP, also had long and sometimes complex dosing schedules.

The main reasons why sampled health workers reported that they did not prescribe AL could be grouped into two broad categories: first, specific failings in the introduction of this policy, and second, more general health system issues. The two key specific failings in AL introduction related to the mixed/unclear and incorrect messages delivered during in-service training, and the availability/continued supply of amodiaquine.

KEMSA continued to supply health facilities with large quantities of non-recommended antimalarials (amodiaquine) resulting in confusion among health workers. This issue had been frequently documented during changes in antimalarial drug policy with, for example, in Tanzania stockpiles of chloroquine leading to implementation problems during SP introduction [34,35]. It is, therefore, essential that plans are put in place well in advance of the introduction of new drugs to ensure that stockpiles of medicines being replaced are removed from the supply chain.

Mixed or ambiguous messages delivered during in-service training had a clearly negative impact on health workers' prescription practices. These included information on the continued efficacy of amodiaquine, compulsory parasitological testing of patients and differential diagnosis of fevers in children. In Makueni District, health workers were universally told that amodiaquine was still effective and could be used in the treatment of uncomplicated malaria. This statement is incorrect, since Makueni was among the first districts in Kenya reporting increased levels of *Plasmodium falciparum *resistance (22%) to amodiaquine as early as in 1997 [36]. Furthermore, such messages clearly contradict the new guidelines, where amodiaquine is not recommended in the treatment of uncomplicated malaria.

With regard to fever in children below five years, some health workers reported that they were told to rule out other causes of fever before prescribing AL. This contradicts the main recommendations stipulated in the new guidelines and accompanying algorithms [5], where presumptive treatment with AL for all childhood fevers in high malaria risk areas is recommended. Inappropriate messages on compulsory malaria diagnosis before prescribing AL are also highlighted in this study. Health workers reported that they were told during the training that testing in patients five years and above was compulsory regardless of the availability of diagnostics. This contradicts the national treatment guidelines which recommend that: "*Patients above 5 years of age with positive test result should be treated with AL, however, where diagnostics are not available, in the absence of other obvious cause of fever AL should be prescribed presumptively for all febrile patients above 5 years of age" *[5]. This confusion was likely to lead to frequent provision of amodiaquine and SP for adult patients.

These problems highlight the importance of the quality of training, a factor rarely captured in quantitative studies on provider behaviour. It appears that in many cases the training was effective in getting across the messages delivered; the problem was that some messages were often inaccurate. This implies a need for greater quality control during the training, perhaps through greater time spent on initial training of trainers, and monitoring of cascade training sessions by more senior staff. It also emphasizes the importance of follow up supervision of health workers in facilities, to monitor their practice, and give them the opportunity to ask further questions and resolve any confusion.

The second set of reasons for non-adherence related to more general health system factors, particularly the workload of health care staff, and the erratic nature of drug supply.

The desired quality of care cannot be fully realized when there is a shortage of health personnel. The Kenyan MoH faces serious shortages of health personnel, especially qualified nurses. This has resulted in unskilled staff working beyond their normal level of competency. The five study districts have two to four doctors, seven clinical officers and 40 to 50 nurses per 100,000 people in each district [37]. In this study, health workers argued that staff shortages resulting in increased workload affected their prescribing decisions. This issue was also investigated in a study on physician practices in the treatment of acute respiratory infection, which highlighted the negative relationship between physician case load and quality of care [28]. AL prescription requires malaria diagnostic procedures where these are available, adequate counseling, direct observation of the first dose, and new reporting procedures, which were argued to be time consuming leading health workers to prefer prescribing amodiaquine. The problems of staff shortages may be addressed to some degree by a recent substantial increase in staff recruitment in Kenya. The MoH, with funding from the Global Fund, the US President's Emergency Plan for AIDS Relief (PEPFAR) and the Clinton Foundation, has recruited 1,428 health workers, comprising mainly registered and enrolled nurses (800), laboratory technologists and technicians (70) and clinical officers (100). The new staff are not limited to delivering HIV services and will provide general primary care services. However, despite these increases, staffing levels remain well below government norms and the long-term future of these staff appointed on short-term contracts is unclear.

Many health workers reported drug stock outs as a key reason for non-prescription of AL. AL supply was reported as erratic in the study districts with at least one stock-out period since the change in treatment policy. However, health workers were only included in these interviews if they were working at health facilities where AL was in stock on the day of the quantitative survey, but they still prescribed it for less than 40% of malaria diagnoses. This indicates that non-prescription may be related to a fear of future stock outs, and therefore the need to conserve AL for priority cases, even when the drug is currently in stock. This is not surprising given the long history and widespread nature of drug stock outs in the Kenyan health system occasioned by poor administration and distribution procedures, and general inefficiencies in the central procurement system [38]. However, several districts in Kenya are currently moving from the 'push' to 'pull' drug delivery system where they order AL based on their consumption requirements, which may help to relieve shortages in the long-term. The perceived need to conserve AL in case of future stock outs led health workers to ration the drug based on their own criteria. They indicated that they would give AL to patients whom they deemed 'deserving'. They described a 'deserving' patient as one who appeared to be more severe, presenting with a high fever/temperature (40°C), headache and joint pains. The influence of perceived severity of illness on health worker' prescribing behaviour has been reported elsewhere [29]. The use of additional criteria such as these, in discordance with national guidelines, is likely to result in missed cases of malaria.

## Conclusion

The introduction of free efficacious ACT in the public health sector in Kenya and other countries has major potential public health benefits for Africa. These however may not be realized if provider prescription practices do not conform to the recommended treatment guidelines. It is, therefore, paramount to understand reasons underlying health workers' non-adherence to recommended treatment guidelines. This study investigated factors affecting health workers' non-prescription of AL.

Some of the key reasons reflect problems in the underlying health system which clearly requires long-term investment such as staffing levels and drug supply. However, other key reasons are specifically related to the process of policy introduction and more amenable to immediate action, such as inappropriate training messages and continued supply of non-recommended antimalarials. The following policy recommendations are implied for both improving AL delivery in Kenya and informing future policy change in Kenya and elsewhere. First it is essential that training, drug supply and supervision work synergistically to ensure concordance in patient management and national treatment guidelines. In particular, there is a need for better quality control of cascade training. Secondly, single, one-off in-service training efforts, that may themselves introduce ambiguities in recommendations, should be augmented with some simple measures of continuous education, dialogue and clinical supervision. Finally, a phase-out plan should be developed for non-recommended antimalarials during the transition period to prevent mixed prescriptions during the introduction of a new antimalarial treatment policy.

## Authors' contributions

BW participated in the study design, development of data collection tools, conducted data collection and analysis, drafted the study report, first and revised versions of the manuscript. DZ participated in the study design, planning and implementation of the study and revision of the manuscript. CAG participated in the study design, development and revision of the study tools and revision of the manuscript. RWS conceived the study, participated in the study design, planning and implementation of the study and manuscript writing.
